# Perioperative Changes in Episodic Memory in Patients Undergoing Brain Tumor Surgery: A Longitudinal Study of Clinical Determinants

**DOI:** 10.3390/cancers18203395

**Published:** 2026-10-20

**Authors:** Kirsten Heckmann, Matthias Simon, Tunc Faik Ersoy

**Affiliations:** Department of Neurosurgery, Bielefeld University, Medical School and University Medical Center OWL, Evangelisches Klinikum Bethel, 33617 Bielefeld, Germany; matthias.simon@evkb.de (M.S.); tunc-faik.ersoy@evkb.de (T.F.E.)

**Keywords:** cognitive function, episodic memory, brain tumors, surgery, clinical risk factors, neuro-oncology

## Abstract

**Background/Objectives**: Episodic memory impairment is common in patients with intracranial tumors and has significant implications for clinical management. Memory is often treated as a unitary construct, and parameter-specific patterns and perioperative changes remain poorly understood. This study aimed to examine different memory parameters, their temporal dynamics, and clinical predictors of impairment. **Methods**: This observational study included 76 patients undergoing resection of supratentorial tumors, assessed preoperatively and at one week and three months postoperatively. Episodic memory was evaluated using a standardized word-list learning paradigm, yielding measures of immediate recall, delayed recall, and recognition. Performance was classified using age-adjusted z-scores, and relevant intraindividual changes were examined. Risk factors for impairment were analyzed using logistic regression. **Results**: Long-term memory measures were more frequently impaired than immediate recall across all time points. Impairment rates increased markedly one week after surgery to as high as 46.5% but returned to baseline levels at three months; however, individual trajectories were heterogeneous. Preoperative impairment was associated with lower functional status. Postoperative memory deficits were strongly associated with preoperative impairment, with older age being additionally associated with transient deficits. **Conclusions**: Memory impairment after brain tumor surgery is dynamic and parameter-specific, with long-term memory particularly vulnerable. Predictor findings are exploratory given limited event numbers and substantial cohort heterogeneity. Although deficits are often transient, high interindividual variability exists, underscoring the need for individualized risk stratification and neuropsychological care. Increased attention should be paid to the vulnerable acute postoperative period, as intact short-term memory may mask impairments.

## Simple Summary

Patients with brain tumors frequently experience memory difficulties that can affect daily functioning and medical decision-making. Memory, however, is not a single ability but comprises different aspects, including immediate recall, delayed recall, and recognition. This study shows that after brain tumor surgery, long-term memory is often impaired, while initial learning remains relatively preserved. Memory problems often worsen shortly after surgery and may not be readily apparent in routine clinical conversations, as patients may appear unimpaired despite meaningful deficits because short-term memory remains intact. Although many patients improve over time, substantial variability remains. Importantly, individuals with pre-existing memory problems or reduced functional status are at particularly high risk. These findings highlight the importance of systematic cognitive assessment and improved communication strategies to better support patients throughout treatment and recovery.

## 1. Introduction

Cognitive impairment is increasingly recognized as a major comorbidity in patients with intracranial tumors, substantially affecting functional independence, return to work, and overall quality of life [1,2,3,4]. Even subtle deficits may prevent otherwise symptom-free long-term survivors from regaining premorbid autonomy and occupational functioning [5]. Cognitive disturbances arise not only from tumor location and mass effect, but also from peritumoral edema, disruption of large-scale brain networks, seizures, and treatment-related factors such as surgery, radiotherapy, systemic therapy, and anticonvulsant medication [6,7]. Indeed, most patients experience neurocognitive impairment at some point during the disease trajectory, with memory and executive functions among the most frequently affected domains [2,8]. Importantly, cognitive dysfunction can be present even in patients with largely preserved everyday functioning [9,10].

Among cognitive domains, episodic memory is of particular clinical relevance in neuro-oncological care. Patients’ ability to encode and retain verbally communicated information is essential during physician–patient consultations and forms the basis for informed decision-making, treatment adherence, and translating medical recommendations into everyday routines [11,12,13]. Its relevance is further underscored by the fact that memory—particularly verbal memory—is one of the most frequently impaired cognitive domains in patients with brain tumors [8].

Despite the high prevalence and clinical relevance of memory deficits, many studies in neuro-oncological populations focus on global cognitive functioning and conceptualize memory as a broad domain alongside attention, executive functions, and processing speed—often operationalized as composite scores. However, cognitive neuroscience models have long emphasized that memory is not a unitary construct but consists of separable functional components supported by partially dissociable neural mechanisms, including encoding, consolidation/storage, and retrieval [14,15,16]. Storage deficits are particularly critical because they reflect an inability to establish stable long-term memory representations, resulting in reduced availability of information, whereas retrieval deficits primarily reflect difficulties in accessing stored information. Although these processes are theoretically distinguishable, their contributions to neuropsychological test performance may overlap. Free recall performance reflects the interplay of encoding, storage, and retrieval processes, while recognition can facilitate access to stored information but also involves retrieval processes [17,18,19]. Nevertheless, a differentiated assessment of memory performance may provide clinically meaningful information beyond global domain scores, allowing for a more precise understanding of patients’ everyday challenges. This is particularly relevant in rehabilitation contexts, where acquiring, retaining, and retrieving new information is crucial for learning compensatory strategies and transferring therapeutic content into daily life [11]. Furthermore, the effect of surgery on different memory parameters remains poorly understood.

Although only a few studies have differentiated between specific memory parameters in brain tumor populations, available evidence suggests that long-term memory measures are more frequently affected than short-term memory [9,20,21]. Delayed recall has been identified as one of the most impaired cognitive functions, even several years after diagnosis [22,23]. To our knowledge, however, evidence from prospective studies combining baseline assessment with a differentiated evaluation of memory performance remains limited. Likewise, associations between clinical characteristics and memory impairments are poorly investigated, limiting individualized risk stratification.

Against this background, the present study aims to provide a differentiated characterization of memory functioning in patients undergoing intracranial tumor resection by separately examining impairments in immediate recall, delayed recall, and recognition, examining their changes across the perioperative course, and exploring clinical risk factors relevant for risk stratification and patient counseling. We thereby aim to contribute to a more fine-grained understanding of memory dysfunction in neuro-oncology and its implications for clinical management and rehabilitation.

## 2. Materials and Methods

### 2.1. Participants and Procedure

For this observational study, patients with a first diagnosis of a supratentorial intracranial tumor who underwent neurosurgical resection between December 2023 and August 2025 were recruited consecutively from the Department of Neurosurgery, Bielefeld University, Medical School and University Medical Center OWL, Evangelisches Klinikum Bethel, Bielefeld. Inclusion criteria were age ≥ 18 years, a sufficient general health condition, adequate hearing, and sufficient language fluency for reliable test administration. Patients with other preexisting major psychiatric or neurological disorders, as well as those with prior cranial radiotherapy or systemic oncological treatment, were excluded. All patients who had suffered a preoperative seizure were started on anticonvulsive medication (29 on levetiracetam, two on lacosamide, and one on both); three patients were already taking pregabalin for neuropathic pain. No other medication with potential sedative effects that might have influenced neuropsychological assessment was administered.

Pertinent clinical data were collected, including age at surgery, gender, Karnofsky-Performance-Score (KPS) pre- and postoperatively and at 3 months, handedness, histopathological diagnoses, number of tumors, tumor load (assessed as single tumor volume or volume of all cerebral (metastatic) deposits in cm^3^), index tumor growth pattern (intra- vs. extra-axial) and hemisphere, index tumor location (frontal, temporal, parietal, occipital, and deep, the latter including the ventricular system, pineal gland, and all tumors with insular involvement; index tumors involving several lobes/regions were assigned to the category harboring the relatively largest part of the tumor), pre- and postoperative seizures, anticonvulsant medication, major complications according to Common Terminology Criteria for Adverse Events (CTCAE III-V) [24], postoperative radiotherapy and systemic treatment.

All patients had adequate preoperative MR imaging, i.e., 3D T1 sequences with and without contrast-enhancing (CE) agents, T2, FLAIR, DWI, and SWI. Tumor volume was calculated using commercially available software (iplanNet, Brainlab SE, version 5.0.1.2, Munich, Germany) quantitatively. For CE glioma, tumor volume was assessed based on the CE-lesion(s) without including the non-enhancing lesion. For non-CE glioma, tumor volume was assessed based on the FLAIR-hyperintense lesion(s). Edema was not assessed.

Verbal memory performance was assessed at three time points: the baseline assessment was carried out during the week before surgery (pre), follow-up assessments were conducted in the acute postoperative phase, approximately one week after surgery (1 w), and in the subacute phase, approximately three months postoperatively (3 m). All assessments were administered by a trained psychologist under standardized conditions. To mitigate confounding effects of situational fatigue, assessments were jointly scheduled with patients and performed only when adequate alertness was reported.

**Figure 1:**
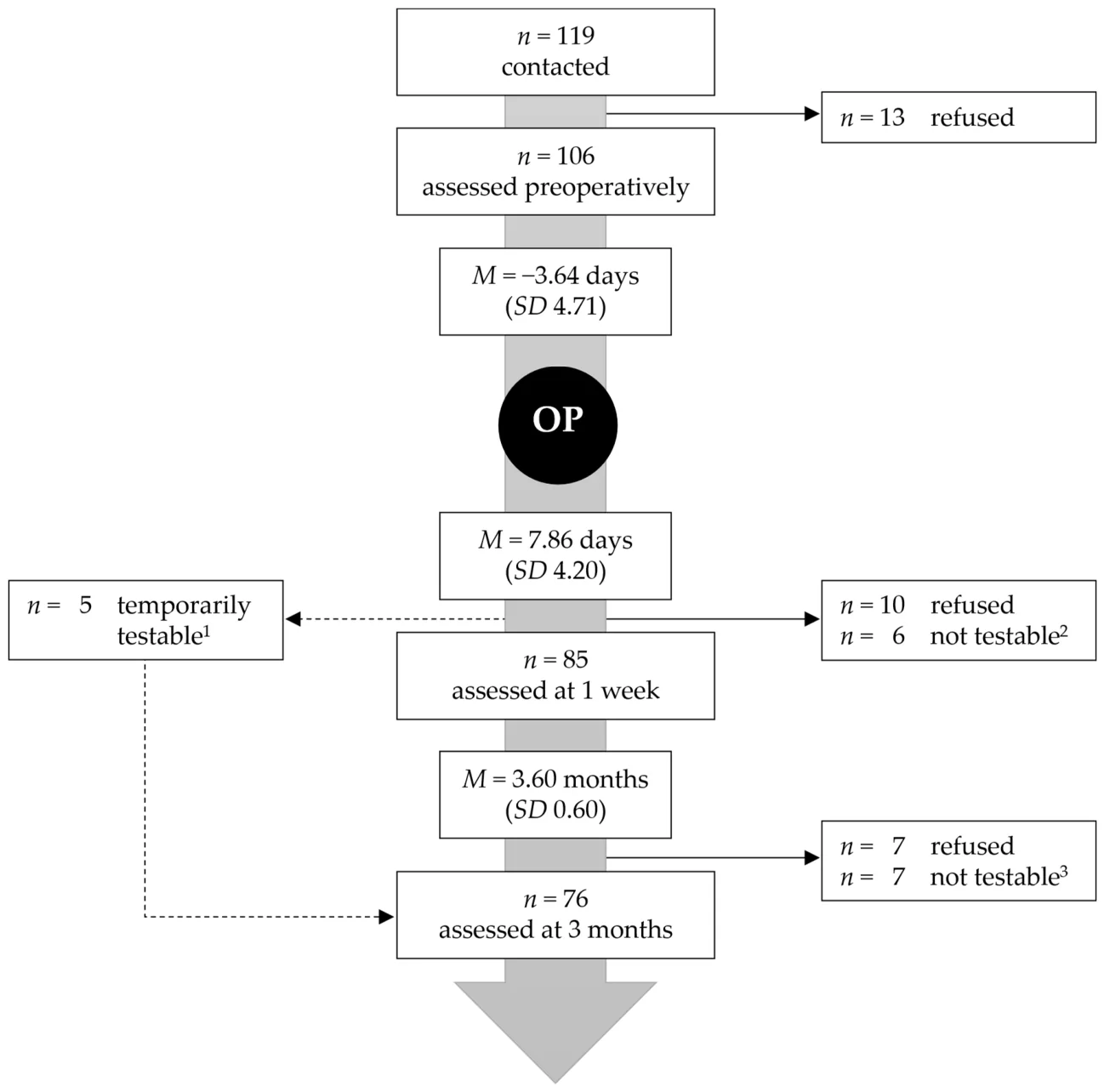
Study details and reasons for attrition. *M* and *SD* refer to 3 m completers. ^1^ The five temporarily untestable patients were able to participate in the 3-month follow-up (indicated by the dashed lines). ^2^ Including death from myocardial infarction (*n* = 1) or surgical complication (*n* = 1). ^3^ Including death from medical complication (*n* = 2, pneumonia and basilar artery occlusion) or tumor progression (*n* = 1).

A total of 76 patients completed both the preoperative and the 3-month follow-up assessments and were included in the longitudinal analyses (hereafter referred to as 3 m completers). Of these, five patients could not be assessed postoperatively for health or emotional reasons but continued with the 3-month follow-up. Figure 1 shows study details and reasons for attrition. Main reasons for refusals were related to subjective physical or emotional strain, or to the logistical burden of attending follow-up appointments.

### 2.2. Materials

Verbal memory performance was assessed by the verbal learning and memory test (VLMT) [25], the German adaptation of the Rey Auditory Verbal Learning Test [26,27]. The VLMT assesses various aspects of episodic memory using a list of 15 words presented auditorily across five consecutive learning trials. Following each trial, participants are asked to recall as many words as possible, providing measures of immediate recall and learning across trials. After the fifth trial, an interference list consisting of 15 novel words is presented and immediately recalled, followed by free recall of the original list. After a 30-min delay, long-term memory processes are assessed by delayed free recall and an additional recognition task, in which participants identify previously learned words from a list of 50 items; recognition performance is corrected for false-positive responses.

The VLMT comprises three parallel versions, which were counterbalanced across the assessments by systematically rotating the starting version across participants.

### 2.3. Statistical Analyses

Based on the study objectives, VLMT recall performance after the first learning trial was used to index a short-term memory process (immediate recall: IR), whereas delayed recall (DR) and recognition (REC) served as long-term memory outcomes [23,27]. Raw scores were converted to age-corrected z-scores based on normative data [25]. According to established neuropsychological conventions, z-values between −1 and +1 were considered to reflect performance within the normal range [27]. To account for variability in commonly applied cut-off values across studies [28,29] and to capture the full spectrum of cognitive performance in the sample, test performance was classified into four categories: impaired (z ≤ −1.5), reduced (−1.5 < z ≤ −1), normal (−1 < z ≤ 1), and above average (z > +1). For subsequent analyses, these categories were collapsed into a binary classification contrasting impaired performance with all other performance levels, applying a z-value of −1.5 as the cut-off. This threshold was chosen to focus on clinically meaningful cognitive dysfunction, in line with recommendations by Sinha et al. [28] and its widespread use in neuro-oncological research [30,31,32,33].

We additionally examined relevant intraindividual change in memory performance to capture substantial changes at the individual level, including declines occurring within normative categories without meeting formal impairment criteria (e.g., due to high baseline performance). Relevant intraindividual change was defined as an increase or decrease of at least three words between baseline and follow-up in delayed recall, and at least four words in recognition performance, based on critical differences provided in the VLMT manual [25]. For immediate recall, the same threshold as for delayed recall was applied.

Baseline characteristics of 3 m completers and non-completers were compared using chi-square tests or Fisher’s exact tests, as appropriate, for categorical variables and independent-samples *t*-tests for continuous variables, with Welch’s *t*-test used in case of unequal variances. For the 3 m completers, independence between categorical demographic and clinical variables was assessed using chi-square tests. Primary analyses focused on changes in memory parameters over time, using Cochran’s Q tests to examine overall changes in the proportion of patients with severe impairment and post-hoc McNemar tests to identify specific pairwise differences.

Exploratory, hypothesis-generating univariable logistic regression analyses were used to examine associations between clinical characteristics and memory impairment. Continuous variables were dichotomized to reduce model complexity and facilitate clinical interpretability. Due to the limited number of events relative to candidate predictors, a two-step analytic strategy was applied to avoid unstable parameter estimates [34]. Univariable logistic regression analyses were first performed to identify potentially relevant predictors of memory impairment. Predictors reaching statistical significance were subsequently entered into exploratory multivariable logistic regression models to explore adjusted associations. Gender and histology were not included due to their strong associations with tumor growth pattern (intra- vs. extra-axial) and because of small subgroup sizes for histology. No multivariable model was performed for immediate recall due to the small number of impaired cases at 3 m (*n* = 5). The threshold for statistical significance was set at *p* < 0.05. All statistical analyses were performed using IBM SPSS Statistics, version 29 (IBM Corp., Armonk, NY, USA).

## 3. Results

### 3.1. Patient Characteristics

Baseline characteristics of 3 m completers and non-completers are presented in Table 1. Compared with non-completers, 3 m completers were younger and more frequently showed a KPS score of 100%. Histological diagnoses also differed between groups. Post-hoc analyses revealed a higher proportion of patients with metastases among non-completers (*p* = 0.030), but no differences for the other histological groups. Of note, a significantly lower proportion of non-completers had preoperative seizures. Regarding memory outcomes, non-completers showed significantly lower performance in delayed recall and, particularly, in recognition, whereas immediate recall performance did not differ between groups. Notably, this was accompanied by a higher impairment rate for recognition, but not for delayed recall.

Complementing the information presented in Table 1, the cohort of 3 m completers was characterized as follows: Mean duration of education was 15.31 ± 3.47 years. Mean KPS was 92.89% ± 11.29% preoperatively, 84.34% ± 15.09% at 1 w, and 88.82% ± 14.04% at 3 m. Most patients were right-handed (89.5%), with only a few left-handed (6.6%) or ambidextrous (3.9%). Histological diagnoses included glioma (23.7% glioblastoma, IDH1-wild type, CNS WHO grade 4; 18.4% IDH-mutant (two IDH-mutant, CNS WHO grade 4; one IDH-mutant and 1p/19q not co-deleted astrocytoma, CNS WHO grade 3; four IDH-mutant and 1p/19q co-deleted oligodendroglioma, CNS WHO grade 3; three IDH-mutant and 1p/19q not co-deleted astrocytoma, CNS WHO grade 2; four IDH-mutant and 1p/19q co-deleted oligodendroglioma, CNS WHO grade 2)), meningioma (38.2%: 24 CNS WHO grade 1, four CNS WHO grade 2, and one CNS WHO grade 3), metastases (9.2%: three bronchial, one breast, one malignant melanoma, one colorectal, one renal cell), and other entities (10.5%: one pilocytic astrocytoma, one chondrosarcoma, one intracranial Ewing sarcoma, one central neurocytoma, one subependymoma, one non-Hodgkin lymphoma, two cavernous hemangiomas). The majority of tumors were located in the frontal lobe (48.7%), followed by temporal (21.1%), parietal (17.1%), occipital (1.3%), and deep-seated (11.8%). Only one IDH-wildtype glioblastoma patient, three IDH-mutant glioma patients, and one meningioma patient had a subtotal removal of their tumor. We observed 10.5% major surgical (three hemorrhages, one ischemia, one hydrocephalus, and three cases of meningitis) and two (2.6%) medical complications; only three patients (3.9%) exhibited a persisting major neurological deficit at discharge. Postoperative treatment was provided according to established guideline-based recommendations for the respective tumor entity and individual clinical indication. During the follow-up period, 44.7% of the patients received radiotherapy and 34.2% received systemic treatment. Notably, no patient received whole-brain radiotherapy.

Chi-square analyses indicated that older participants, compared to younger participants, more frequently had right-hemispheric (64.9% vs. 40.5%, *p* = 0.036) and extra-axial tumors (50.0% vs. 26.3%, *p* = 0.034), whereas all other clinical factors were unrelated to age.

Compared to women, men more frequently had a preoperative KPS score below 100% (46.3% vs. 22.9%, *p* = 0.033), intra-axial tumors (80.5% vs. 40.0%, *p* < 0.001), and a larger tumor load (61.0% vs. 37.1%, *p* = 0.038).

### 3.2. Group-Level Changes in Memory Performance

**Figure 2:**
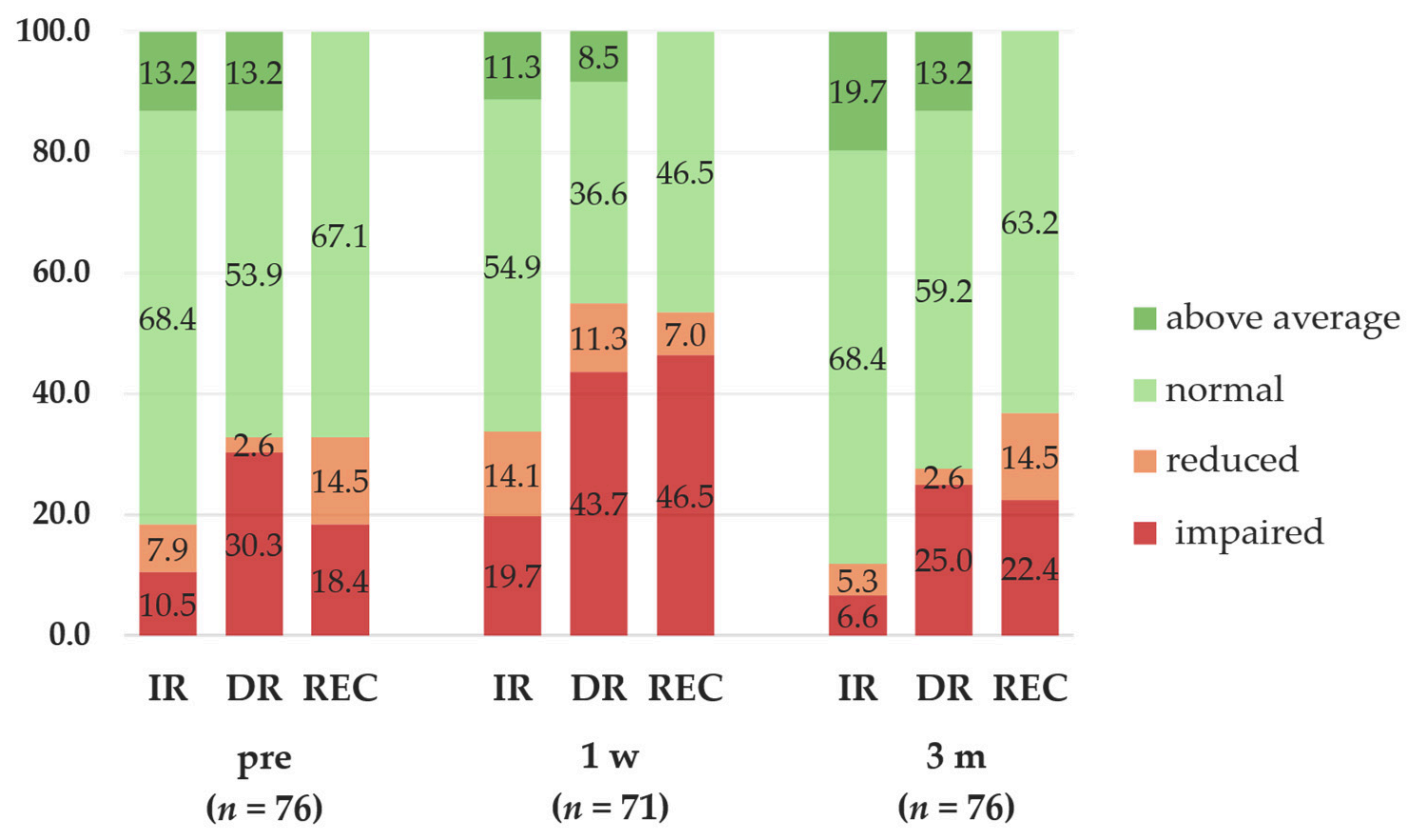
Distribution of memory performance across parameters for 3 m completers. IR = immediate recall, DR = delayed recall, REC = recognition. In the VLMT recognition measure, above-average performance cannot be obtained.

**Figure 3:**
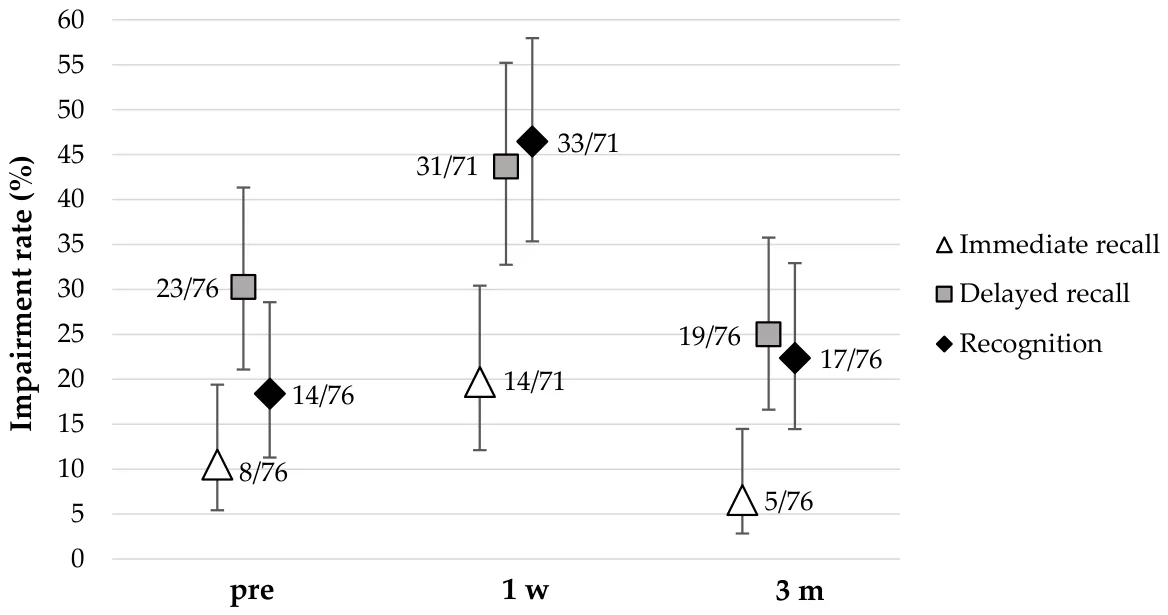
Impairment rates across memory parameters and assessment time points for 3 m completers. Error bars represent 95% Wilson score confidence intervals; numbers adjacent to markers indicate *n*/*N*.

Table 2 summarizes age-adjusted z-scores and intraindividual changes in memory performance across assessment time points for 3 m completers. Overall, mean performance declined from preoperative assessment to 1 w and improved again by 3 m, with substantial interindividual variability in both memory performance and change. Given this variability, categorical outcomes were analyzed across parameters and assessment time points to better characterize the distribution of memory performance (Figure 2) and changes in impairment rates over time (Figure 3).

Across all time points, delayed recall and recognition were more frequently impaired than immediate recall, indicating relative preservation of short-term memory. Impairment rates changed significantly over time for all memory parameters, with the strongest effect for recognition (Cochran’s Q tests, *n* = 71; IR: Q(2) = 9.00, *p* = 0.011; DR: Q(2) = 9.75, *p* = 0.008; REC: Q(2) = 25.15, *p* < 0.001). Pairwise McNemar tests showed a significant increase in recognition impairment from pre to 1 w (*n* = 71, *p* < 0.001), followed by a significant decrease from 1 w to 3 m (*n* = 71, *p* < 0.001). Delayed recall followed a similar pattern, although the increase from pre to 1 w did not remain significant after applying a Bonferroni-corrected significance level of α = 0.017 (pre–1 w: *n* = 71, *p* = 0.049; 1 w–3 m: *n* = 71, *p* = 0.004). Notably, the increase in impairment rates in immediate recall from pre to 1 w (*n* = 71, *p* = 0.109) was not significant. None of the memory parameters differed significantly between pre and 3 m in categorical terms (*n* = 76; IR: *p* = 0.453; DR: *p* = 0.454; REC: *p* = 0.549). Yet, severe long-term memory deficits were evident in roughly one quarter of patients. These findings remained robust after excluding patients who suffered from any major complication (*n* = 9), showing the same transient postoperative increase in impairment.

### 3.3. Intraindividual Changes in Memory Performance

**Figure 4:**
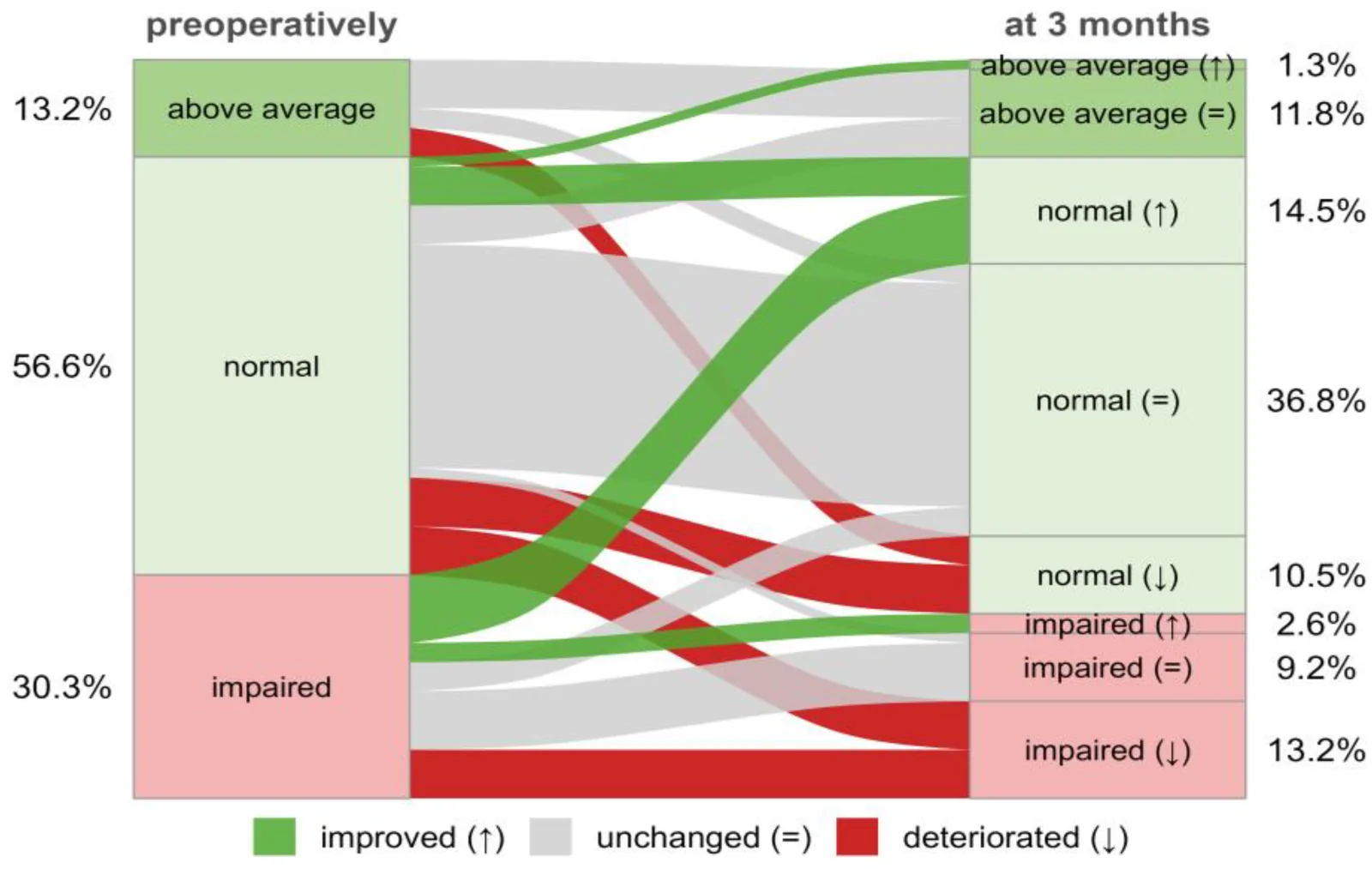
Alluvial-linked bar charts depicting relevant intraindividual change from preoperative assessment to 3-month follow-up in delayed recall.

**Figure 5:**
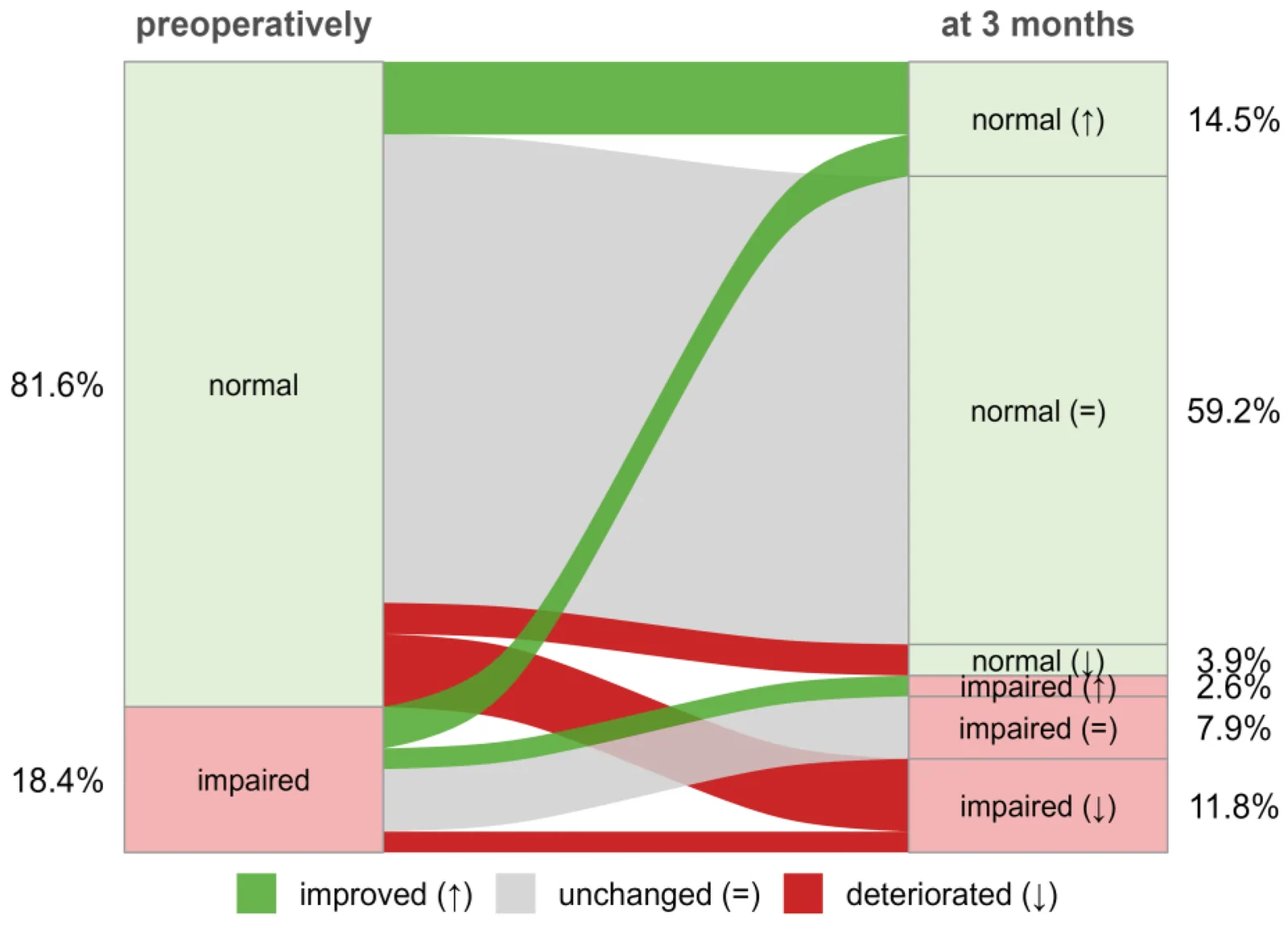
Alluvial-linked bar charts depicting relevant intraindividual change from preoperative assessment to 3-month follow-up in recognition. In the VLMT recognition measure, above-average performance cannot be obtained.

**Figure A1:**
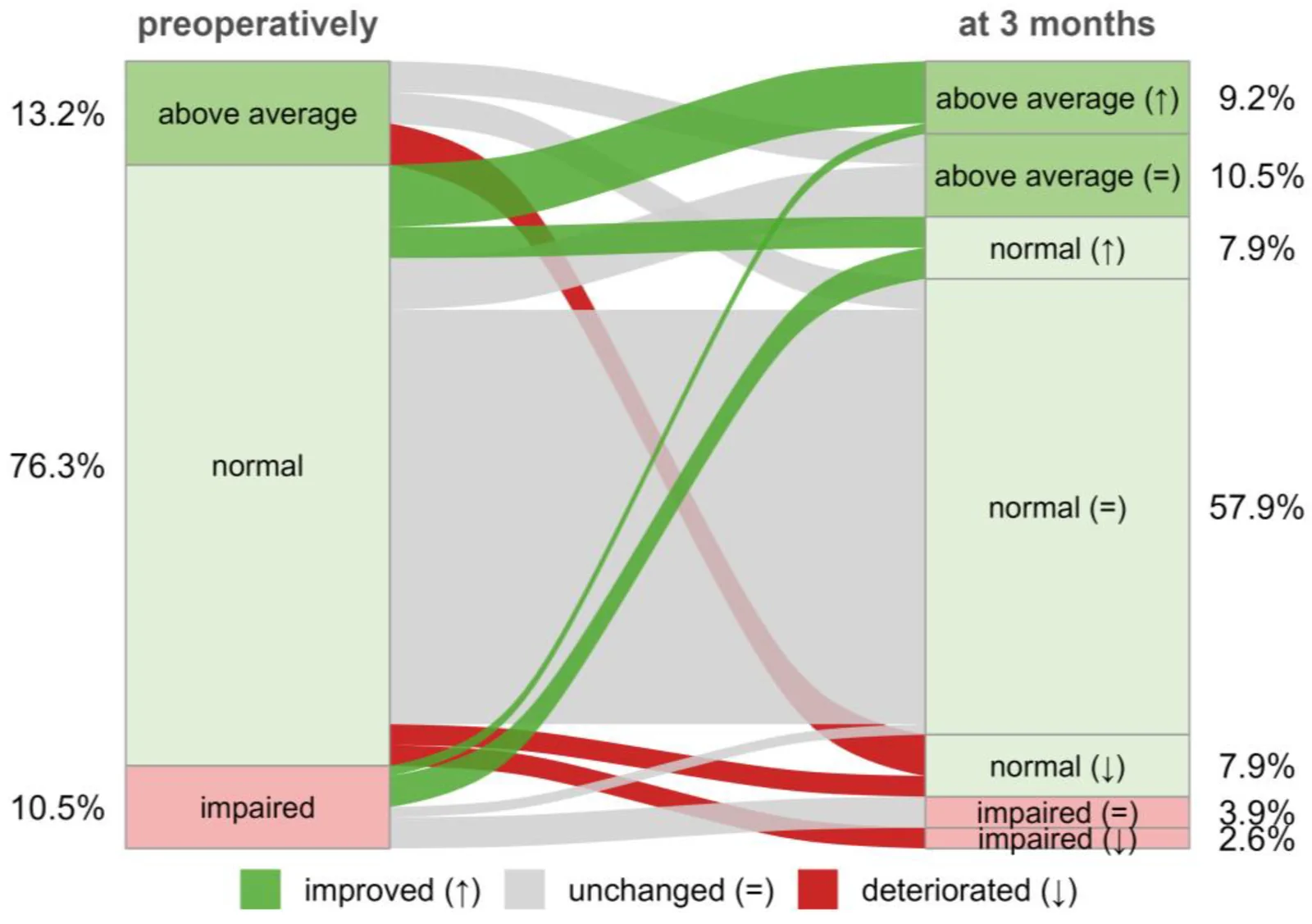
Alluvial-linked bar charts depicting relevant intraindividual change from preoperative assessment to 3-month follow-up in immediate recall.

While group-level analyses indicated a return to approximately baseline levels 3 months after surgery, analyses of intraindividual change revealed substantial heterogeneity, particularly for delayed recall and recognition, as demonstrated in Figure 4 and Figure 5. The alluvial-linked bar charts combine information on transitions between normative performance categories with relevant intraindividual changes in test performance. This allows the identification of patients who show relevant improvement or deterioration while remaining within the same performance category, as well as patients who move between categories without showing a relevant change in individual performance (i.e., whose performance was close to a category threshold). The corresponding figure for immediate recall is presented in Appendix A (Figure A1).

In delayed recall, relevant intraindividual decline was more frequent than improvement overall (23.7% vs. 18.4%), primarily driven by patients who were unimpaired preoperatively: 24.5% (13/53) of those unimpaired at baseline declined, with 9.4% (5/53) transitioning into the impaired range. By contrast, within the preoperatively impaired subgroup, more patients improved than declined (39.1% (9/23) vs. 21.7% (5/23)), although a shift towards greater impairment severity was also evident. At 3 m, five patients (6.6%) were unable to recall any word in delayed recall, compared with one patient (1.3%) preoperatively.

For recognition, deterioration was less frequent, with broadly comparable rates of improvement and decline (17.1% vs. 15.8%). As observed for delayed recall, improvements outnumbered further deterioration among impaired patients (42.9% (6/14) vs. 14.3% (2/14)). Importantly, however, severe recognition failure became more common: seven patients (9.2%) selected more incorrect than correct items at 3 m, resulting in negative recognition scores, compared with two patients (2.6%) at baseline.

In immediate recall, relevant intraindividual deterioration at 3 m was least frequent (10.5%), whereas improvement was observed more often (17.1%).

Table 3 summarizes clinical characteristics of individuals showing relevant intraindividual change in delayed recall and recognition. Overall, patients who improved and those who declined showed largely comparable clinical profiles. Descriptively, patients with a KPS below 100% exhibited relevant intraindividual changes more frequently than those with a KPS of 100%, particularly in recognition, albeit in both directions. Relevant intraindividual improvements in both parameters were more frequently observed in patients with larger compared to smaller tumors, whereas patients with gliomas more often showed relevant decline than patients with other histologies. However, given the small subgroup sizes, no statistically significant subgroup differences were observed.

Importantly, intraindividual trajectories among patients who suffered from any major complication (*n* = 9) were largely comparable to those observed in the overall cohort. In delayed recall, six patients within this subgroup remained stable, one improved, and two declined between the preoperative assessment and 3 m. In recognition, seven patients remained stable, one improved, and one declined. Overall, impairment rates in this subgroup were similar at baseline and 3 m (DR: 1 vs. 2 patients, REC: 1 vs. 0 patients).

### 3.4. Associations with Clinical Parameters

Univariable logistic regression analyses were conducted to examine associations between clinical parameters and memory impairment in delayed recall and recognition across time points (Table 4 and Table 5). Even subtle preoperative functional impairment (i.e., KPS < 100%) was strongly associated with preoperative memory deficits. Although poorer preoperative functional health remained associated with impairments in both parameters at 1 w—and for recognition even at 3 m, preoperatively existing memory impairment appeared to be much more influential for memory outcome.

Other preoperative factors associated with memory impairment included male gender—and higher tumor load for recognition. Although older age (>58.3 yrs.) was relevant for the acute postoperative phase, its effect on memory outcome did not persist in the long term. For delayed recall, male gender remained associated with impairment at 3 m; additional associations were found for left-hemispheric lateralization and intra-axial growth pattern at this stage. For recognition, male gender and higher tumor load were associated with impairments at 3 m.

Additional analyses were conducted for immediate recall (see Appendix B). Due to the small number of patients with impairments—particularly at 3 m—analyses were limited by statistical power and unstable estimates. No clear associations with clinical parameters were observed for preoperative impairment in immediate recall. In contrast, postoperative impairment in immediate recall was significantly associated with preoperative impairment at both postoperative time points. At 3 m, all five patients with immediate recall impairment were male and had intra-axial tumors.

**Figure 6:**
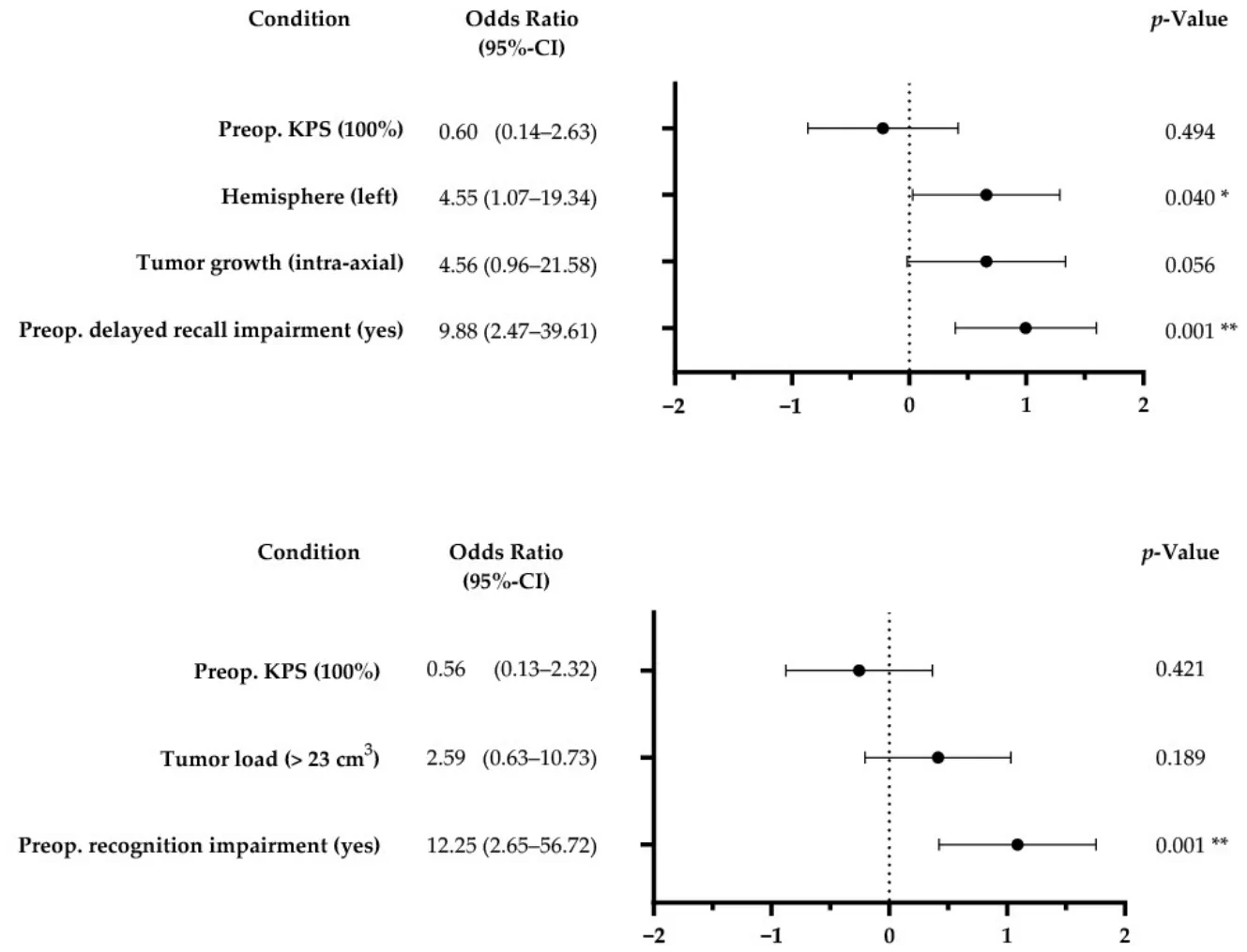
Exploratory multivariable logistic regression analysis of univariable significant variables for delayed recall (above, *n* = 74 excluding two patients with bilateral tumors) and recognition impairments (below, *n* = 76) at 3 m. Categories in parentheses indicate the category for which the odds ratio is reported. X-axes are shown on a log scale. * *p* < 0.05; ** *p* < 0.01.

Exploratory multivariable analyses including all significant variables from the univariable models (without gender and histology) suggested that impairments in delayed recall remained associated with pre-existing delayed recall deficits (OR = 25.58, 95% CI [4.35, 150.42], *p* < 0.001) and older age (OR = 11.06, 95% CI [2.23, 54.98], *p* = 0.003) at 1 w (*n* = 71), while a preoperative KPS of 100% was no longer significantly associated with lower odds of impairment (OR = 0.38, 95% CI [0.10, 1.44], *p* = 0.154). At 3 m, preoperative delayed recall deficits (*p* = 0.001) and left-hemispheric lateralization (*p* = 0.040) remained associated with delayed recall impairment in the multivariable model (Figure 6). For recognition, preoperatively (*n* = 76), only a KPS score of 100% remained associated with lower odds of impairment (OR = 0.12, 95% CI [0.03, 0.48], *p* = 0.003), whereas higher tumor load was no longer associated with recognition impairment (OR = 3.36, 95% CI [0.77, 14.57], *p* = 0.106). At both postoperative assessments, preoperative recognition impairment was the only variable that remained significant after multivariable adjustment. More specifically, pre-existing recognition impairment was associated with higher odds of postoperative recognition impairment (OR = 16.09, 95% CI [1.79, 144.42], *p* = 0.013) at 1 w (*n* = 71), whereas a preoperative KPS of 100% (OR = 0.56, 95% CI [0.16, 1.89], *p* = 0.346) and older age (OR = 2.74, 95% CI [0.92, 8.21], *p* = 0.071) did not retain statistical significance. Corresponding estimates for the 3 m multivariable model are presented in Figure 6.

## 4. Discussion

In this paper, we present preliminary findings from a longitudinal study investigating three key parameters of episodic memory in patients undergoing surgery for intracranial tumors, and their relation to clinical parameters. As functional outcome becomes increasingly important, neuropsychological impairments are receiving greater attention in neurosurgery.

### 4.1. Temporal Dynamics

Consistent with previous literature, substantial memory impairments were evident both preoperatively and postoperatively [22,35,36,37,38,39,40,41,42]. All memory parameters showed significant changes over time, with pronounced deterioration—particularly in long-term memory—at 1 w, followed by marked recovery. By 3 m, impairment rates largely returned to baseline, in line with reports of transient postoperative decline and subsequent recovery [2,20,39,43,44]. Thus, at group level, our findings suggest that deterioration in memory function is primarily a short-term rather than a longer-term outcome after surgery.

At the individual level, trajectories revealed substantial heterogeneity. Nearly one in five patients showed relevant intraindividual improvement at 3 m. While this pattern may reflect genuine improvement in a subset of patients, alternative explanations should also be considered. These include regression to the mean as well as practice/test-familiarity effects. In addition, potential floor effects—i.e., the limited ability to register further decline in patients with very low baseline performance—may have inflated the apparent proportion of improvements. This finding may still be informative for surgical decision-making, particularly in patients for whom surgery is often approached cautiously because of advanced age, extensive intra-axial tumors, and pre-existing neurocognitive impairment. Conversely, a substantial number of patients exhibited decline relative to their individual baseline, particularly in delayed recall. Given the lower overall impairment rates at 3 m compared to baseline, this suggests that clinically relevant changes frequently occur within normative ranges and may be missed by categorical classifications. This aligns with prior work demonstrating that apparent group-level recovery can obscure individual decline [43]. The pronounced heterogeneity observed in intraindividual trajectories—where patients with broadly similar clinical characteristics may nonetheless improve or worsen—highlights the value of perioperative neuropsychological assessment for detecting subtle yet meaningful changes and providing timely targeted cognitive support. These findings underscore the importance of integrating specialized neuropsychological expertise into routine clinical care.

### 4.2. Memory Parameter Differentiation

A key strength of this study is the fine-grained differentiation between immediate recall, delayed recall, and recognition, moving beyond aggregated memory scores and the increasing reliance on computerized paradigms that primarily assess recognition-based responses and place fewer demands on retrieval.

In line with prior studies, impairments predominantly affected long-term memory measures, while short-term memory, as reflected by immediate recall, remained relatively preserved [20,23]. However, in contrast to Durand et al. [23], recognition impairment in our cohort was as frequent as delayed recall impairment at 3 m. While this does not necessarily imply impaired storage, as reduced discrimination between previously learned and new information and potential response bias also need to be considered [45], it may still indicate more extensive impairment of memory performance than impaired free recall alone. This difference likely reflects the earlier assessment in our study, potentially capturing ongoing recovery and network reorganization [46]. The continued presence of recognition-related deficits may reflect continued vulnerability of underlying long-term memory processes beyond the acute phase, with recovery potentially occurring only over longer intervals, as suggested by studies with extended follow-up [44,47,48,49].

### 4.3. Acute Postoperative Phase

A central contribution of this study is the characterization of the acute postoperative phase, which has received comparatively little attention despite its high clinical relevance and the risk of meaningful cognitive dysfunction going undetected. At this stage, nearly half of the patients exhibited deficits in both delayed recall and recognition, including complete failure and increased false-positive responses during recognition—patterns suggesting more fundamental impairment [11,50]. Given that this phase coincides with the communication of complex and highly consequential therapeutic information, such impairments may compromise patients’ ability to retain and integrate critical medical information, potentially undermining treatment adherence and informed shared decision-making [51]. Future studies should more closely examine the impact of memory impairment on everyday functioning and quality of life using ecologically valid measures, such as adherence to follow-up care.

Particularly during the acute postoperative phase, older patients and those with preoperative functional or memory impairments appeared especially vulnerable, independently of tumor characteristics. Importantly, preserved short-term memory may mask these impairments in routine interactions. This risk is further amplified in patients who tend to endorse closed questions, potentially creating a misleading impression of preserved memory functioning. To improve detection, ward rounds should probe recall of prior conversations, instructions, or recent events, rather than relying solely on yes-or-no questions. For vulnerable patients in particular, verbal information should be supplemented with written materials, and the involvement of family members or caregivers is strongly recommended.

### 4.4. Role of Clinical Parameters

We assessed associations with a number of clinical parameters that have been linked to cognitive performance before, despite inconsistencies across studies [52].

Preoperatively, functional status showed the strongest association with memory impairment, supporting the established association between lower KPS and poorer cognitive performance [1,31,53,54]. Even subtle functional limitations were linked to deficits in both delayed recall and recognition, whereas tumor characteristics were not associated after adjusting for KPS. Postoperatively, pre-existing memory impairment was consistently associated with postoperative impairment at both 1 w and 3 m, in line with prior studies [55,56]. However, this did not imply further deterioration after surgery in patients with preoperative memory impairment. Rather, as demonstrated in Section 3.3., within this group, relevant intraindividual improvement was more frequently observed than further deterioration. Nonetheless, some patients with preoperative impairments experienced additional decline, manifesting as very severe memory deficits. When interpreting changes in this subgroup, potential floor effects should be considered, since very low baseline scores can limit the measurable range of additional decline.

Age was associated with impairment only at 1 w, not at 3 m, suggesting an increased vulnerability in older patients during the acute phase that does not appear to extend into the longer term. Because scores were benchmarked against age-adjusted norms [25], this acute effect exceeds expected age-related decline. Given the importance of preoperative KPS as noted above, functional health may possibly be more relevant than age when considering patients for surgery.

At 3 m, left-hemispheric lateralization was the only tumor characteristic that remained associated with delayed recall impairment after multivariable adjustment, whereas no corresponding association was observed for recognition impairment. Extending prior reports linking left-hemispheric tumors to overall memory performance [20,23,43,57,58], our data point to a preferential disruption of free access to information that remained accessible under recognition conditions. The absence of a preoperative association between left-hemispheric lateralization and delayed recall further suggests that this aspect of memory performance may be particularly vulnerable postoperatively. Due to the small sample size, we were unable to adequately investigate the effect of tumor location on memory.

Gender differences in delayed recall at 3 m likely reflect underlying imbalances in tumor load, tumor growth characteristics, and baseline functional status, potentially compounded by a general female advantage in verbal episodic memory [59]. Although other predictors showed domain-specific patterns, they did not retain significance in multivariable models. Notably, preoperatively, neither metastases nor gliomas were associated with higher rates of memory impairment compared with meningiomas, consistent with reports of substantial cognitive deficits in meningioma cohorts [60,61,62]. We did observe higher impairment rates at 3 m in the univariable analyses among glioma patients. Unfortunately, due to the small histological subgroup sizes, we cannot draw robust conclusions regarding their effect on different memory parameters. Higher impairment rates in high- versus low-grade tumors have, however, been reported repeatedly [36,39,41,63], suggesting that biological aggressiveness and growth dynamics may be more informative than anatomical classification alone. Larger, biologically stratified cohorts are needed to robustly disentangle the independent effects of relevant tumor characteristics.

No associations were observed for seizure history, consistent with previous findings [23], potentially reflecting the use of modern, cognitively more favorable anticonvulsant medication such as levetiracetam [64], which was the standard medication in our cohort.

### 4.5. Limitations

Generalizability is limited by selective sampling and attrition. The cohort of 3 m completers was characterized by high functional status (mean KPS > 90%), exceeding that of typical brain tumor populations [65,66], and by a comparatively younger mean age (54.3 years), but was comparable to prior neuropsychological studies with similar constraints [39,67]. The relatively high level of formal education (mean 15.3 years) may further indicate selective participation of more highly educated patients. Attrition analyses additionally revealed that non-completers were older, less frequently had a preoperative KPS of 100%, and showed lower baseline performance in delayed recall and recognition, with a higher prevalence of recognition impairment. These findings suggest that clinical or cognitive impairment may have reduced the likelihood of completing follow-up assessments. Consequently, more severely affected patients may be underrepresented, potentially leading to an underestimation of the prevalence of memory impairment [67]. In addition, the overrepresentation of right-hemispheric and extra-axial tumors among older patients in the 3 m completers cohort may have influenced age-related findings.

Potential influences of perioperative medications other than anticonvulsants, fatigue, and mood on memory performance cannot be excluded, particularly during the acute postoperative phase. However, potential contributions of medication or fatigue do not diminish the clinical relevance of the observed memory impairments, as these factors are part of the clinical situation at this stage. Potential practice/test-familiarity effects despite the use of parallel test versions should also be considered. Notably, such effects would be expected to favor better rather than poorer performance and therefore would not account for the pronounced increase in memory impairment observed at 1 w. Postoperative memory outcomes may have been influenced by the extent of resection and residual tumor. At 3 m, additional influences of postoperative treatment as confounders for malignant tumors (i.e., radiotherapy and systemic treatment) and CNS recurrence cannot be excluded. Finally, the predictor analyses were exploratory and involved multiple testing. Their interpretation is further constrained by the overall sample size and the resulting limited absolute number of patients meeting impairment criteria, which increases the risk of overfitting in multivariable models. The findings should therefore be interpreted cautiously and require confirmation in larger independent samples. A further limitation is the dichotomization of continuous variables such as age and tumor volume. Moreover, the number of patients showing relevant intraindividual change was insufficient to support adequately powered predictor analyses of distinct postoperative trajectories. Such analyses are an important next step towards identifying patients at increased risk of relevant deterioration and those more likely to show improvement in the medium and longer term, thereby enabling trajectory-informed risk stratification and more individualized preoperative counseling. Furthermore, the absence of a nonsurgical control group limits the extent to which the observed postoperative trajectories can be attributed specifically to surgery. The limited follow-up period of 3 months further precludes conclusions regarding longer-term recovery or persistence of memory impairment. Lastly, we lack information on the impact of memory deficits in daily life settings, such as adherence to oncological treatment or return to work.

## 5. Conclusions

Postoperative memory function in brain tumor patients undergoing resection is time-dependent and differs across memory parameters. At the group level, memory impairments were largely already present before surgery, with the postoperative increase in impairment being temporary and impairment rates returning to preoperative levels at 3 m. At the individual level, most patients showed no evidence of persistent deterioration at 3 m. In the acute postoperative period, intact short-term memory should not be taken as evidence of preserved long-term memory. At this stage, particularly older patients and those with even subtle functional limitations may have difficulty retaining verbally conveyed information over time. Therefore, proactive communication strategies and written supplements are warranted to ensure that essential information remains accessible. The increased vulnerability of older patients appears to be limited to the acute postoperative phase. Beyond the observed association between left-hemispheric lateralization and delayed recall impairment, larger, biologically stratified cohorts are needed to enable more accurate identification of patients at risk of persistent postoperative memory impairment and relevant intraindividual deterioration. We aim to address these questions in a subsequent analysis of our ongoing study, including 12-month follow-up.

Finally, our data highlight that group-level changes do not necessarily reflect intraindividual trajectories. This underscores the need for individualized cognitive assessment as part of routine neuro-oncological care and for a more consistent integration of neuropsychological findings into pre- and postoperative counseling and shared clinical decision-making.

## Figures and Tables

**Table 1:** Comparison of baseline characteristics between 3 m completers and 3 m non-completers.

		3 m Completers (*n* = 76)		3 m Non-Completers (*n* = 30)		Group Comparison
Variable	Category	*n*	%	*n*	%	*p*
Gender	male female	41 35	53.9% 46.1%	14 16	46.7% 53.3%	0.499
KPS preop.	KPS < 100% KPS 100%	27 49	35.5% 64.5%	20 10	66.7% 33.3%	**0.004**
Hemisphere ^1^	right left	39 35	52.7% 47.3%	14 16	46.7% 53.3%	0.577
Location	frontal temporal parieto-occipital deep	37 16 14 9	48.7% 21.1% 18.4% 11.8%	13 5 9 3	43.3% 16.7% 30.0% 10.0%	0.630
Tumor growth	extra-axial intra-axial	29 47	38.2% 61.8%	13 17	43.3% 56.7%	0.624
Histology	meningioma glioma metastases other	29 32 7 8	38.2% 42.1% 9.2% 10.5%	13 9 80	43.3% 30.0% 26.7% 0.0%	**0.036 ** ^2^
Seizures preop.	no yes	44 32	57.9% 42.1%	24 6	80.0% 20.0%	**0.033**
IR impairment	no yes	68 8	89.5% 10.5%	27 3	90.0% 10.0%	1.000 ^2^
DR impairment	no yes	53 23	69.7% 30.3%	20 10	66.7% 33.3%	0.758
REC impairment	no yes	62 14	81.6% 18.4%	17 13	56.7% 43.3%	**0.008**
**Variable**	**Unit**	***M***	***SD***	***M***	***SD***	***p***
Age	years	54.34	14.25	67.05	7.70	**<0.001**
Tumor load	cm^3^	34.80	34.11	33.06	26.90	0.803
IR performance	z-score	−0.22	1.01	−0.47	0.89	0.238
DR performance	z-score	−0.55	1.35	−1.15	1.23	**0.036**
REC performance	z-score	−0.57	1.16	−1.48	1.24	**<0.001**

IR: immediate recall; DR: delayed recall; REC: recognition. Statistically significant *p*-values (*p* < 0.05) are highlighted in bold. ^1^ Percentages for 3 m completers are based on *n* = 74 because of two patients with bilateral tumors. ^2^ Fisher’s exact test.

**Table 2:** Age-adjusted z-scores and intraindividual changes in memory performance across assessment points for 3 m completers.

Memory Parameter	Pre (*n* = 76) M (SD) [95% CI]	1 w (*n* = 71) M (SD) [95% CI]	3 m (*n* = 76) M (SD) [95% CI]	Pre–1 w (*n* = 71) ΔM (SD) [95% CI]	1 w–3 m (*n* = 71) ΔM (SD) [95% CI]	Pre–3 m (*n* = 76) ΔM (SD) [95% CI]
Immediate recall (IR)	−0.22 (1.01) [−0.45, 0.01]	−0.54 (1.22) [−0.82, −0.25]	0.00 (1.08) [−0.25, 0.25]	−0.28 (1.19) [−0.56, 0.00]	0.55 (1.11) [0.29, 0.81]	0.22 (1.17) [−0.05, 0.49]
Delayed recall (DR)	−0.55 (1.35) [−0.86, −0.24]	−1.24 (1.40) [−1.57, −0.91]	−0.56 (1.35) [−0.87, −0.25]	−0.64 (1.09) [−0.90, −0.38]	0.70 (1.02) [0.46, 0.94]	−0.01 (1.13) [−0.26, 0.25]
Recognition (REC)	−0.57 (1.16) [−0.83, −0.30]	−1.41 (1.42) [−1.74, −1.07]	−0.75 (1.25) [−1.03, −0.46]	−0.81 (1.03) [−1.06, −0.57]	0.67 (1.06) [0.42, 0.92]	−0.18 (1.02) [−0.41, 0.05]

Values are age-adjusted z-scores and are presented as mean (SD) [95% CI]. Changes (Δ) represent within-person differences between assessment time points, with positive values indicating improvement and negative values indicating deterioration.

**Table 3:** Rates of relevant intraindividual change from baseline to 3 months in delayed recall and recognition by clinical characteristics for 3 m completers.

Characteristics		Delayed Recall		Recognition	
		Deteriorated *n* (%)	Improved *n* (%)	Deteriorated *n* (%)	Improved *n* (%)
Age	≤58.3 yrs (*n* = 38) >58.3 yrs (*n* = 38)	7 (18.4%) 11 (28.9%)	7 (18.4%) 7 (18.4%)	5 (13.2%) 7 (18.4%)	6 (15.8%) 7 (18.4%)
Gender	male (*n* = 41) female (*n* = 35)	10 (24.4%) 8 (22.9%)	9 (22.0%) 5 (14.3%)	7 (17.1%) 5 (14.3%)	8 (19.5%) 5 (14.3%)
KPS preop.	KPS < 100% (*n* = 27) KPS 100% (*n* = 49)	7 (25.9%) 11 (22.4%)	6 (22.2%) 8 (16.3%)	6 (22.2%) 6 (12.2%)	9 (33.3%) 4 (8.2%)
Tumor load	≤23 cm^3^ (*n* = 38) >23 cm^3^ (*n* = 38)	8 (21.1%) 10 (26.3%)	3 (7.9%) 11 (28.9%)	5 (13.2%) 7 (18.4%)	4 (10.5%) 9 (23.7%)
Hemisphere	right (*n* = 39) left (*n* = 35)	7 (17.9%) 11 (31.4%)	8 (20.5%) 5 (14.3%)	5 (12.8%) 7 (20.0%)	8 (20.5%) 5 (14.3%)
Location	frontal (*n* = 37) temporal (*n* = 16) parieto-occipital (*n* = 14) deep (*n* = 9)	8 (21.6%) 5 (31.3%) 3 (21.4%) 2 (22.2%)	8 (21.6%) 3 (18.8%) 1 (7.1%) 2 (22.2%)	5 (13.5%) 3 (18.8%) 2 (14.3%) 2 (22.2%)	7 (18.9%) 4 (25.0%) 0 (0.0%) 2 (22.2%)
Tumor growth	extra-axial (*n* = 29) intra-axial (*n* = 47)	5 (17.2%) 13 (27.7%)	5 (17.2%) 9 (19.1%)	2 (6.9%) 10 (21.3%)	4 (13.8%) 9 (19.1%)
Histology	meningioma (*n* = 29) glioma (*n* = 32) metastases (*n* = 7) other (*n* = 8)	5 (17.2%) 11 (34.4%) 1 (14.3%) 1 (12.5%)	5 (17.2%) 6 (18.8%) 2 (28.6%) 1 (12.5%)	2 (6.9%) 7 (21.9%) 0 (0.0%) 3 (37.5%)	4 (13.8%) 6 (18.8%) 2 (28.6%) 1 (12.5%)
Seizures preop.	no (*n* = 44) yes (*n* = 32)	11 (25.0%) 7 (21.9%)	9 (20.5%) 5 (15.6%)	4 (9.1%) 8 (25.0%)	9 (20.5%) 4 (12.5%)
Memory impairment preop. ^1^	no (*n* = 53)/(*n* = 62) yes (*n* = 23)/(*n* = 14)	13 (24.5%) 5 (21.7%)	5 (9.4%) 9 (39.1%)	10 (16.1%) 2 (14.3%)	7 (11.3%) 6 (42.9%)

Values are *n* (%). Percentages are based on the category-specific denominators shown in the table. ^1^ Separated values indicate the respective sample sizes for delayed recall/recognition.

**Table 4:** Univariable logistic regression analyses predicting impairment in delayed recall for 3 m completers, with odds ratios (OR), 95% confidence intervals, and *p*-values.

Predictor	Comparison ^1,2^	Pre (*n* = 76)		1 w (*n* = 71)		3 m (*n* = 76)	
		OR (95% CI)	*p*	OR (95% CI)	*p*	OR (95% CI)	*p*
Age	≤58.3 yrs (*n* = 38) vs. >58.3 yrs (*n* = 38)	1.13 (0.43–3.02)	0.803	4.54 (1.65–12.49)	**0.003**	1.53 (0.54–4.36)	0.428
Gender	male (*n* = 41) vs. female (*n* = 35)	0.29 (0.10–0.86)	**0.025**	0.43 (0.16–1.14)	0.091	0.09 (0.02–0.41)	**0.002**
KPS preop.	KPS < 100% (*n* = 27) vs. KPS 100% (*n* = 49)	0.21 (0.07–0.59)	**0.003**	0.21 (0.07–0.59)	**0.003**	0.38 (0.13–1.11)	0.077
Tumor load	≤23 cm^3^ (*n* = 38) vs. >23 cm^3^ (*n* = 38)	1.88 (0.69–5.09)	0.215	1.94 (0.75–5.02)	0.175	2.04 (0.70–5.95)	0.190
Hemisphere	right (*n* = 39) vs. left (*n* = 35)	1.51 (0.56–4.12)	0.418	1.02 (0.39–2.64)	0.972	3.25 (1.07–9.84)	**0.037**
Location	frontal (*n* = 37) vs. temporal (*n* = 16) parieto-occipital (*n* = 14) deep (*n* = 9)	1.87 (0.53–6.58) 0.85 (0.19–3.73) 3.89 (0.86–17.68)	0.332 0.828 0.079	1.36 (0.40–4.59) 1.50 (0.41–5.51) 3.50 (0.74–16.60)	0.619 0.541 0.115	1.95 (0.51–7.44) 1.17 (0.26–5.34) 3.43 (0.73–16.17)	0.329 0.840 0.119
Tumor growth	extra-axial (*n* = 29) vs. intra-axial (*n* = 47)	2.17 (0.74–6.38)	0.158	2.00 (0.74–5.41)	0.172	4.47 (1.17–17.06)	**0.028**
Histology	meningioma (*n* = 29) vs. glioma (*n* = 32) metastases (*n* = 7) other (*n* = 8)	2.62 (0.84–8.22) 1.53 (0.24–9.95) 1.28 (0.20–8.01)	0.098 0.654 0.793	2.14 (0.73–6.32) 2.67 (0.49–14.56) 1.20 (0.23–6.19)	0.167 0.257 0.827	6.74 (1.69–26.91) 1.44 (0.13–16.42) 1.24 (0.11–13.82)	**0.007** 0.767 0.862
Seizures preop.	no (*n* = 44) vs. yes (*n* = 32)	0.84 (0.31–2.28)	0.729	0.77 (0.30–2.00)	0.595	1.33 (0.47–3.78)	0.592
Memory impairment preop.	no (*n* = 53) vs. yes (*n* = 23)	—	—	12.46 (3.55–43.73)	**<0.001**	10.18 (3.12–33.27)	**<0.001**

Statistically significant *p*-values (*p* < 0.05) are highlighted in bold. ^1^ The first category listed for each predictor served as the reference category. For binary comparisons, the first category was coded as 0 and the second as 1. The dependent variable was coded as 0 = unimpaired and 1 = impaired. ^2^ Numbers of participants are given for the whole sample. Five patients could not be assessed at 1 w for health or emotional reasons.

**Table 5:** Univariable logistic regression analyses predicting impairment in recognition for 3 m completers, with odds ratios (OR), 95% confidence intervals, and *p*-values.

Predictor	Comparison ^1,2^	Pre (*n* = 76)		1 w (*n* = 71)		3 m (*n* = 76)	
		OR (95% CI)	*p*	OR (95% CI)	*p*	OR (95% CI)	*p*
Age	≤58.3 yrs (*n* = 38) vs. >58.3 yrs (*n* = 38)	3.04 (0.86–10.73)	0.085	3.43 (1.29–9.13)	**0.014**	3.05 (0.95–9.75)	0.060
Gender	male (*n* = 41) vs. female (*n* = 35)	0.15 (0.03–0.71)	**0.017**	0.57 (0.22–1.48)	0.250	0.18 (0.05–0.70)	**0.013**
KPS preop.	KPS < 100% (*n* = 27) vs. KPS 100% (*n* = 49)	0.10 (0.02–0.38)	**<0.001**	0.25 (0.09–0.71)	**0.009**	0.20 (0.06–0.64)	**0.006**
Tumor load	≤23 cm^3^ (*n* = 38) vs. >23 cm^3^ (*n* = 38)	4.75 (1.21–18.74)	**0.026**	2.41 (0.92–6.27)	0.072	4.42 (1.29–15.18)	**0.018**
Hemisphere	right (*n* = 39) vs. left (*n* = 35)	0.56 (0.17–1.85)	0.339	1.04 (0.40–2.69)	0.933	1.34 (0.45–3.97)	0.596
Location	frontal (*n* = 37) vs. temporal (*n* = 16) parieto-occipital (*n* = 14) deep (*n* = 9)	3.75 (0.85–16.50) 2.25 (0.43–11.66) 2.36 (0.36–15.50)	0.080 0.334 0.372	1.75 (0.52–5.87) 2.04 (0.56–7.50) 3.50 (0.74–16.60)	0.365 0.282 0.115	2.13 (0.49–9.30) 3.56 (0.84–15.06) 3.20 (0.60–17.10)	0.313 0.085 0.174
Tumor growth	extra-axial (*n* = 29) vs. intra-axial (*n* = 47)	2.65 (0.67–10.45)	0.164	1.14 (0.44–2.99)	0.788	2.39 (0.70–8.21)	0.166
Histology	meningioma (*n* = 29) vs. glioma (*n* = 32) metastases (*n* = 7) other (*n* = 8)	2.89 (0.69–12.17) 3.47 (0.46–26.37) 1.24 (0.11–13.82)	0.148 0.230 0.862	1.34 (0.47–3.84) 0.94 (0.18–5.02) 0.75 (0.15–3.79)	0.586 0.940 0.728	2.84 (0.78–10.34) 2.50 (0.36–17.57) 0.89 (0.09–9.33)	0.114 0.357 0.925
Seizures preop.	no (*n* = 44) vs. yes (*n* = 32)	1.04 (0.32–3.35)	0.950	0.64 (0.25–1.65)	0.350	1.30 (0.44–3.84)	0.639
Memory impairment preop.	no (*n* = 62) vs. yes (*n* = 14)	—	—	24.05 (2.93–197.47)	**0.003**	19.64 (4.84–79.75)	**<0.001**

Statistically significant *p*-values (*p* < 0.05) are highlighted in bold. ^1^ The first category listed for each predictor served as the reference category. For binary comparisons, the first category was coded as 0 and the second as 1. The dependent variable was coded as 0 = unimpaired and 1 = impaired. ^2^ Numbers of participants are given for the whole sample. Five patients could not be assessed at 1 w for health or emotional reasons.

## Data Availability

The raw data supporting the conclusions of this article will be made available by the authors on request.

## Abbreviations

The following abbreviations are used in this manuscript:

VLMT	Verbal Learning and Memory Test
KPS	Karnofsky-Performance-Score
pre	Preoperative assessment
1 w	Assessment 1 week after surgery
3 m	Assessment 3 months after surgery

## Appendix B

**Table A1:** Univariable logistic regression analyses predicting impairment in immediate recall for 3 m completers, with odds ratios (OR), 95% confidence intervals, and *p*-values.

Predictor	Comparison ^1,2^	Pre (*n* = 76)		1 w (*n* = 71)		3 m (*n* = 76)	
		OR (95% CI)	*p*	OR (95% CI)	*p*	OR (95% CI)	*p*
Age	≤58.3 yrs (*n* = 38) vs. >58.3 yrs (*n* = 38)	0.30 (0.06–1.57)	0.153	1.38 (0.43–4.49)	0.592	1.54 (0.24–9.80)	0.646
Gender	male (*n* = 41) vs. female (*n* = 35)	0.35 (0.07–1.88)	0.222	0.66 (0.20–2.22)	0.505	— ^3^	—
KPS preop.	KPS < 100% (*n* = 27) vs. KPS 100% (*n* = 49)	0.91 (0.20–4.14)	0.902	0.97 (0.29–3.30)	0.965	2.31 (0.25–21.79)	0.464
Tumor load	≤23 cm^3^ (*n* = 38) vs. >23 cm^3^ (*n* = 38)	8.36 (0.97–71.65)	0.053	0.90 (0.28–2.90)	0.860	4.35 (0.46–40.90)	0.198
Hemisphere	right (*n* = 39) vs. left (*n* = 35)	1.55 (0.32–7.46)	0.586	5.56 (1.37–22.51)	**0.016**	1.73 (0.27–11.04)	0.560
Location	frontal (*n* = 37) vs. temporal (*n* = 16) parieto-occipital (*n* = 14) deep (*n* = 9)	5.14 (0.43–61.32) 6.00 (0.50–72.21) 18.00 (1.60–202.95)	0.195 0.158 **0.019**	2.42 (0.52–11.28) 1.32 (0.21–8.25) 5.80 (1.08–31.11)	0.262 0.768 **0.040**	— ^3^ — ^3^ — ^3^	— — —
Tumor growth	extra-axial (*n* = 29) vs. intra-axial (*n* = 47)	4.90 (0.57–42.08)	0.147	2.67 (0.67–10.61)	0.164	—^3^	—
Histology	meningioma (*n* = 29) vs. glioma (*n* = 32) metastases (*n* = 7) other (*n* = 8)	4.00 (0.42–38.07) 4.67 (0.26–85.55) 9.33 (0.72–120.41)	0.228 0.299 0.087	2.55 (0.59–11.08) 6.00 (0.88–40.87) 1.14 (0.10–12.78)	0.213 0.067 0.817	— ^3^ — ^3^ — ^3^	— — —
Seizures preop.	no (*n* = 44) vs. yes (*n* = 32)	0.81 (0.18–3.65)	0.781	1.48 (0.46–4.78)	0.514	2.17 (0.34–13.83)	0.411
Memory impairment preop.	no (*n* = 68) vs. yes (*n* = 8)	—	—	20.63 (3.54–120.34)	**<0.001**	19.80 (2.66–147.32)	**0.004**

Statistically significant *p*-values (*p* < 0.05) are highlighted in bold. ^1^ The first category listed for each predictor served as the reference category. For binary comparisons, the first category was coded as 0 and the second as 1. The dependent variable was coded as 0 = unimpaired and 1 = impaired. ^2^ Numbers of participants are given for the whole sample. Five patients could not be assessed at 1 w for health or emotional reasons. ^3^ Odds ratios could not be reliably estimated due to sparse data and quasi-complete separation. All five cases of impairment occurred in male patients with intra-axial tumors.
